# A Rare Case Report of Ruptured Cyst Presenting With Acute Meningitis in an Untreated Neurocysticercosis

**DOI:** 10.7759/cureus.13222

**Published:** 2021-02-08

**Authors:** Hammam Shereef, Mahad Ahmed, Mohamedanwar Ghandour, Gino Tapia-Zegarra

**Affiliations:** 1 Internal Medicine, Beaumont Health, Dearborn, USA; 2 Internal Medicine/Nephrology, Wayne State University Detroit Medical Center, Detroit, USA; 3 Infectious Diseases Consultants, San Antonio, USA

**Keywords:** neurocysticercosis, meningitis, subarachnoid

## Abstract

Neurocysticercosis (NCC) rarely presents as acute meningitis; however, when it does, it is not distinguishable clinically from other more common infectious etiologies. Here, we report a case of NCC presenting as acute meningitis, which also highlights the importance of brain MRI imaging rather than CT where possible, the need to include MRI of the spine in patients with the subarachnoid disease, and the limitations of NCC antigen detection assay in cerebrospinal fluid when used in ventriculoperitoneal shunt specimens. A prolonged course of albendazole, praziquantel, and corticosteroids led to the resolution of our patient's NCC.

## Introduction

Neurocysticercosis (NCC) is the most common parasitic central nervous system (CNS) infection in human beings [1]. It follows the ingestion of the larval stage (*Cysticercus cellulosae*) of the swine tapeworm *Taenia solium*. NCC is the most common cause of secondary seizure disorder for which patients may need to be on long-term anti-epileptic medication [2]. Approximately 50 million persons worldwide are estimated to have cysticercosis infection, and as per WHO, around 50 thousand die annually because of NCC and seizures related to NCC.

Cysticercosis is endemic in many regions of Central and South America, sub-Saharan Africa, India, and Asia. A review of the national database found 18,584 hospitalizations of NCC in the USA between 2003 and 2012. Among them, 74% were Hispanics [3].

NCC can present as a parenchymal infection (ParNCC), which is the most common form and carries a better prognosis than the less common extraparenchymal disease (ExPNCC). Seizures are the main presenting feature of ParNCC in up to 80%, whereas ExPNCC can manifest as an intraventricular form followed by the subarachnoid, spinal, and ocular NCC [4].

## Case presentation

**Figure 1:**
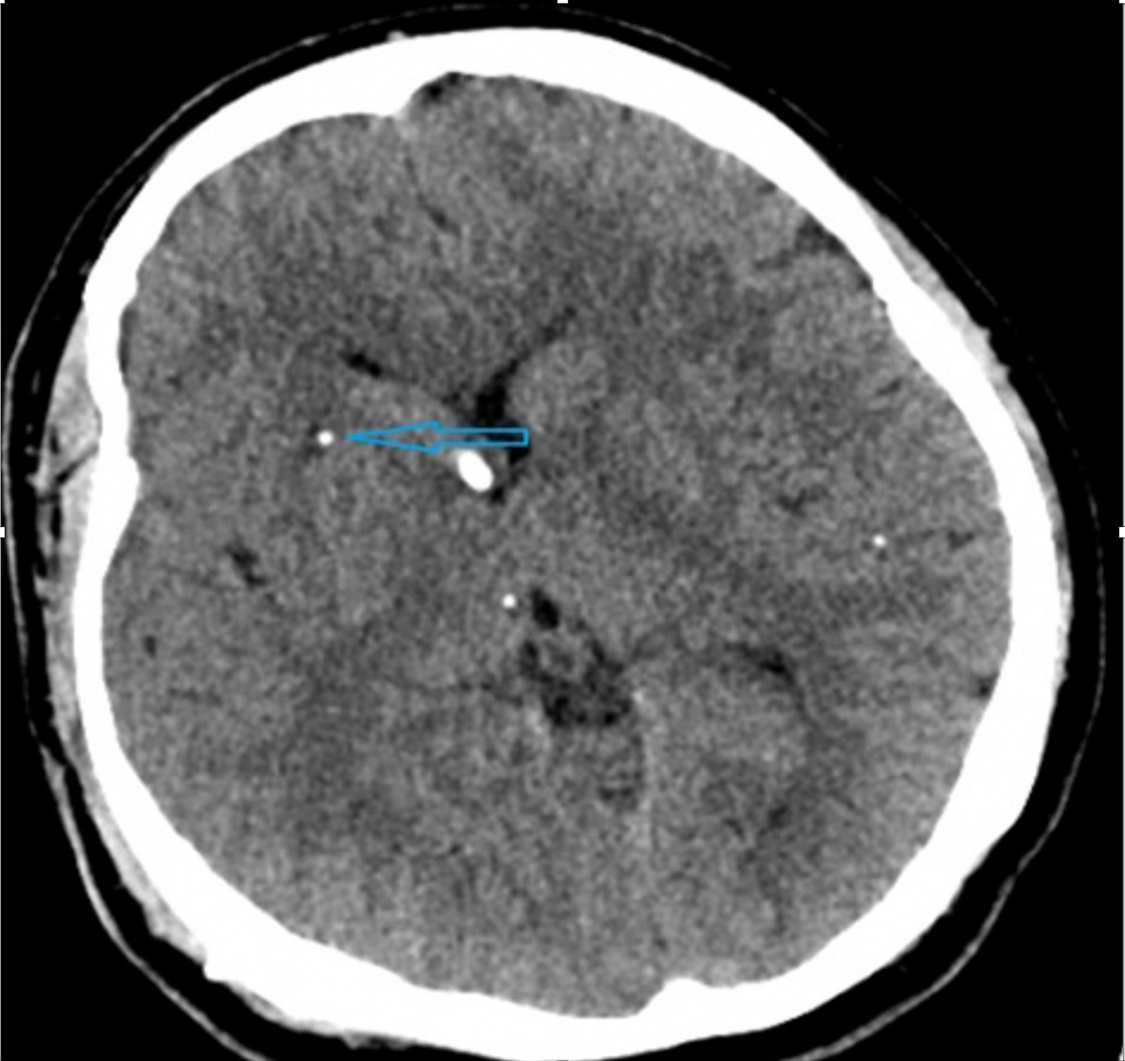
CT of the head showing calcifications (blue arrow).

We report the case of a 31-year-old male from rural Mexico who immigrated to the USA in 2001 with no prior medical and surgical histories; he initially presented to our hospital in 2009 with obtundation and no report of seizures, fever, or other complaints. The patient worked in farms in rural Mexico, where he had eaten undercooked pork. Workup at that time revealed hydrocephalus with multiple calcified brain lesions on brain CT with no cysts or edema (Figure 1) with positive serum *T. solium* serology and negative blood QuantiFERON® (Qiagen, Hilden, Germany). The patient was treated with a ventriculoperitoneal (VP) shunt placement. CT of the brain lesions did not show active cysticercosis; thus, no anti-parasitic treatment was given at initial presentation.

In July 2017, the patient presented with chief complaints of headache and fever of one-day duration but no other symptoms. Physical examination was unremarkable, including no meningeal signs or focal neurologic deficit and unremarkable funduscopic examination. Non-contrast CT of the head showed stable calcified brain lesions since 2009 and no cysts/edema or new changes. Lumbar puncture (LP) revealed cerebrospinal fluid (CSF) white blood cell (WBC) count of 3,320/cumm (81% neutrophils, 11% lymphocytes, no eosinophils), red blood cell (RBC) of 80/cumm, glucose of 9 mg/dL (glucose in blood of 198 mg/dL) protein of 687 g/dL (serum protein of 6.4; normal range: 6.0-8.0 g/dL). On the same day of LP, VP shunt CSF analysis revealed WBC of 20 (34% neutrophils, 59% lymphocytes, no eosinophils), RBC of 16,900 (traumatic tap reported), glucose of 124 mg/dL, and protein of 343 g/d. CSF from VP shunt grew *Propionibacterium acnes*. CSF from LP blood cultures did not reveal any organisms, and CSF polymerase chain reactions (PCRs) were negative for herpes simplex, varicella-zoster, cytomegalovirus, adenovirus, and enterovirus. CSF NCC antigen in the blood (done at the CDC) and CSF (from VP shunt) were negative; however, EITB (enzyme-linked immunoelectrotransfer blot) NCC antibody was positive in CSF and serum. 

**Figure 2:**
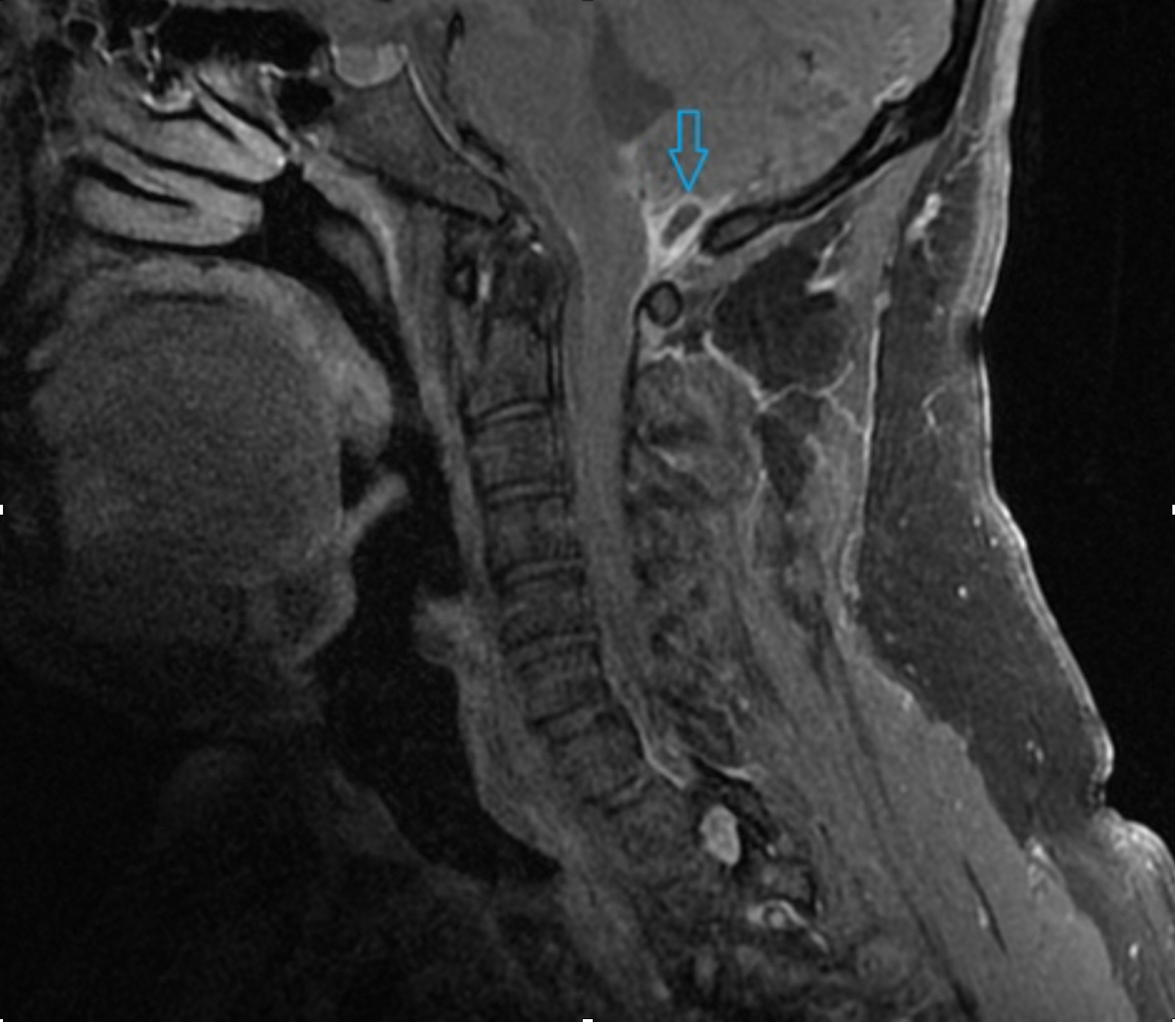
T1-weighted image with contrast showing leptomeningeal enhancement dorsal surface of the upper cervical cord (blue arrow).

**Figure 3:**
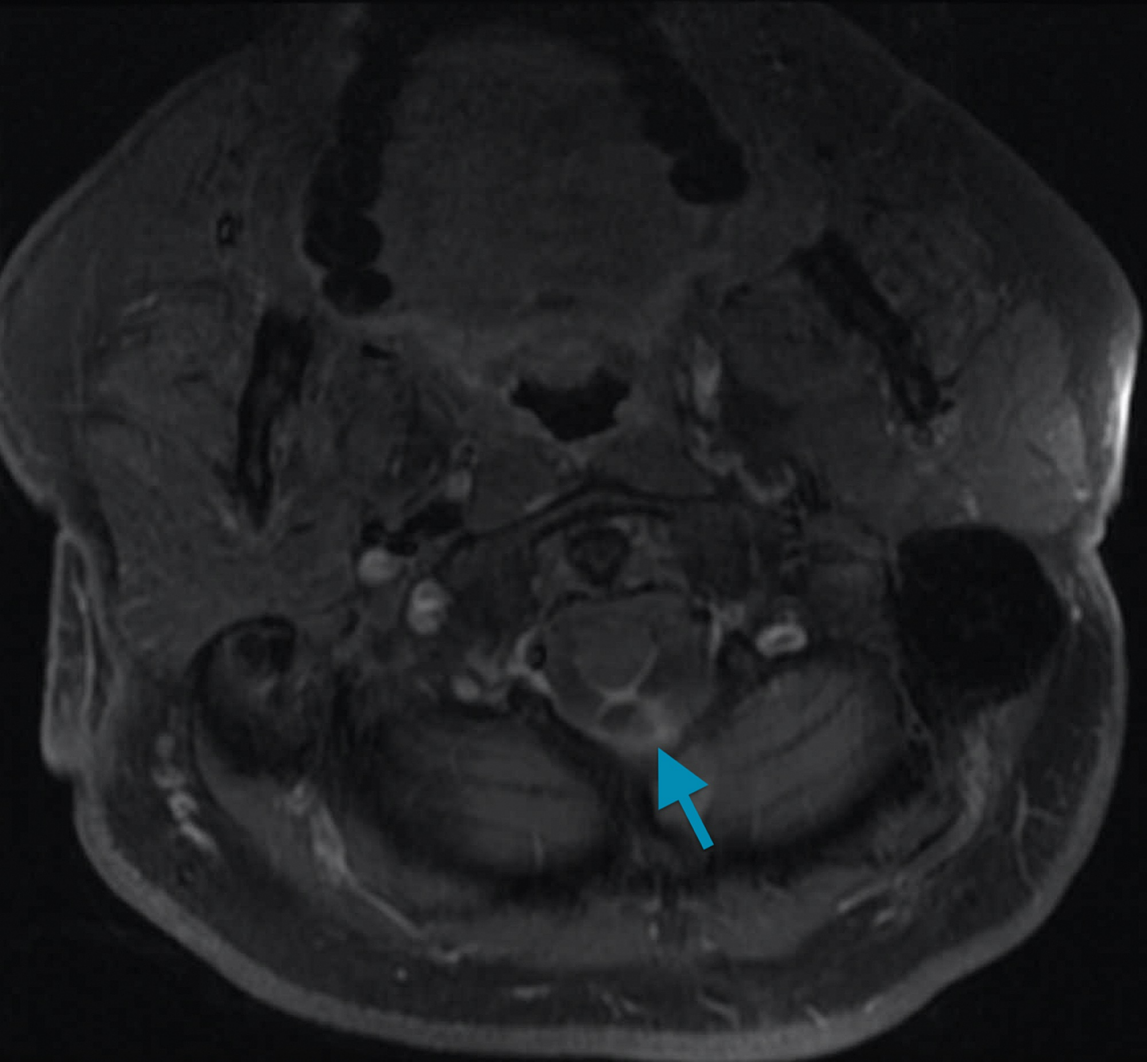
Enhancement dorsal to the cord at the level of the foramen magnum (blue arrow).

The patient completed two weeks of empiric intravenous ceftriaxone and vancomycin and became asymptomatic within two days of his hospitalization. Pertinent findings from cervical, thoracic, lumbar, and brain contrast MRIs (Figures 2, 3) showed peripherally enhancing cystic lesion measuring 1 cm with ill-defined mural nodule measuring 5 mm identified along the dorsal surface of upper cervical cord at the level of the foramen magnum. Local leptomeningeal enhancement was present along the dorsal surface of the upper cervical cord extending inferiorly to the C1 level and superiorly into the foramen of Magendie. The small extra-axial fluid collection extends posteriorly into the inferior left posterior fossa along the inferior-medial margin of the left cerebellar hemisphere, directly posterior to the left cerebellar tonsil measuring 13 x 6 mm, without marginal enhancement, which felt to be reactive to ruptured cyst inflammation. These findings were compatible with NCC cyst rupture.

Follow-up

The patient completed six months of praziquantel 1,800 mg three times daily and albendazole 400 mg twice daily, along with long-term prednisone slowly tapered with good tolerability and resolution of the enhancing cystic lesion along the upper cervical cord at the level of the foramen magnum, leptomeningeal enhancement, and inflammatory signs per follow-up brain MRI performed in October 2017. These MRI findings remained unchanged without inflammatory changes on repeat brain MRI performed in January 2018 upon discontinuation of anti-parasitic therapy, and the patient remains asymptomatic to date.

## Discussion

It is estimated that the NCC incidence in the United States range from 0.2 to 0.6 cases per 100,000 general population and 1.5-5.8 cases per 100,000 Hispanics [5]. ExPNCC (subarachnoid, meningeal, and intraventricular space) is less common than parenchymal infection (ParNCC). The manifestations of ExPNCC range from asymptomatic lesions to meningitis, stroke, and hydrocephalus [6,7]. Subarachnoid NCC can present with communicating hydrocephalus, meningitis, stroke, or focal neurologic deficits [7]. A study published in 2004 reported that 91% of NCC cases presented with parenchymal infection, subarachnoid cysts in 2%, ventricular cysts in 6%, and hydrocephalus in 16% of NCC cases. [8]. In contrast, a case series between (1997-2005) from Houston, Texas, United States, included a total of 111 patients, of whom 60 patients (54%) of had parenchymal disease, 22 patients (20%) with intraventricular involvement, 13 patients (12%) had subarachnoid disease, and 13 (12%) had calcifications only; moreover, two patients only had hydrocephalus, and one patient developed ocular cysticercosis [9]. Moreover, a case series from New Mexico reported that 30% of cases were of ExPNCC [10]. Cysticercal meningitis (CM), which represents the host inflammatory response to the parasite, is usually chronic and occurs in less than 8% of adult NCC cases [6,8]. There have been few cases of NCC presenting as acute meningitis [8,11], although it is probably an underreported presentation. It is unclear if any reported cases were due to a ruptured NCC cyst, which was present in our patient.

The recommended EITB assay (the most reliable test for the detection of antibodies specific for T. solium antigens in serum or CSF) has a specificity approaching 100% and a sensitivity varying from 70% to 90% depending on the number of cysts present [12,13]. However, A positive cysticercosis antibody does not distinguish between previous or active infection. Besides, in endemic regions, there is a positive background seroprevalence of 5-20% [14]. Therefore, parasite antigen detection assays seem useful for both diagnosis (which are very specific for viable infection and its detection in CSF is specific to CNS infection) and treatment monitoring (parasite antigens typically fall after successful treatment). Antigen assays are more sensitive when done in CSF, which, in turn, can be falsely negative mainly due to a single intracranial cysticercus [13,15]. Standardized antigen detection assays are not commercially available in the United States currently [7].

Treatment of subarachnoid NCC may include prolonged courses of albendazole or combinations of praziquantel and albendazole until radiological resolution of viable cysticerci in conjunction with corticosteroids use to downregulate the immune response associated with anti-parasitic use [7,16,17].

## Conclusions

There is a clinical dilemma when a patient develops acute meningitis in the setting of subarachnoid NCC and the presence of a VP shunt device, which is always concerning for device infection. In terms of diagnostic criteria, our patient would have been diagnosed with device infection (by P. acnes) by which he would have warranted device removal as recommended by the Infectious Diseases Society of America (IDSA). However, based on our patient's radiologic and serologic diagnostic criteria met for NCC as suggested by IDSA, quick improvement without device removal made the diagnosis of shunt infection improbable, leading to the diagnosis of a ruptured NCC cyst as the only pathologic process in our patient and P. acnes as being a CSF specimen contaminant and therefore avoiding shunt removal. All patients with hydrocephalus and risk factors for NCC should have a thorough evaluation for NCC including brain MRI, if possible, to look for additional cysticerci as those cases can also have asymptomatic involvement with additional lesions in the parenchyma, subarachnoid space, or ventricles with important prognostic and monitoring implications. Given its strong association with spinal involvement, all patients with intracranial subarachnoid disease should also undergo an MRI of the spine. If the NCC antigen detection assay is used in a patient with a VP shunt, it is best to obtain the CSF specimen from an LP, particularly in subarachnoid NCC, where circulating antigens are almost always present.
